# Supplementary material for: Reputation risk during dishonest social decision-making modulates anterior insular and cingulate cortex activity and connectivity
Source: Commun Biol. 2023 Apr 29;6:475. doi: 10.1038/s42003-023-04827-w (PMC10148859; doi:10.1038/s42003-023-04827-w)
Supplement: Supplementary file 1 — Supplemental Material [file 42003_2023_4827_MOESM1_ESM.pdf]

GLM 1

No reputation		Reputation		Instruction	
Lie	Truth	Lie	Truth	Lie	Truth
1	2	3	4	5	6

GLM 2

No Reputation				Reputation				Instruction			
Unfavourable		Favourable		Unfavourable		Favourable		Unfavourable		Favourable	
SG-Lie	OG-Truth	OG-Lie	SG-Truth	SG-Lie	OG-Truth	OG-Lie	SG-Truth	SG-Lie	OG-Truth	OG-Lie	SG-Truth
1	2	3	4	5	6	7	8	9	10	11	12

Figure S1. Visual representation of first level regressors: Top) GLM1: 6 regressors for overall contrasts of lie vs. truth in the 3 main conditions. Bottom) GLM2: 12 regressors for contrasts of different types of lies and truths.

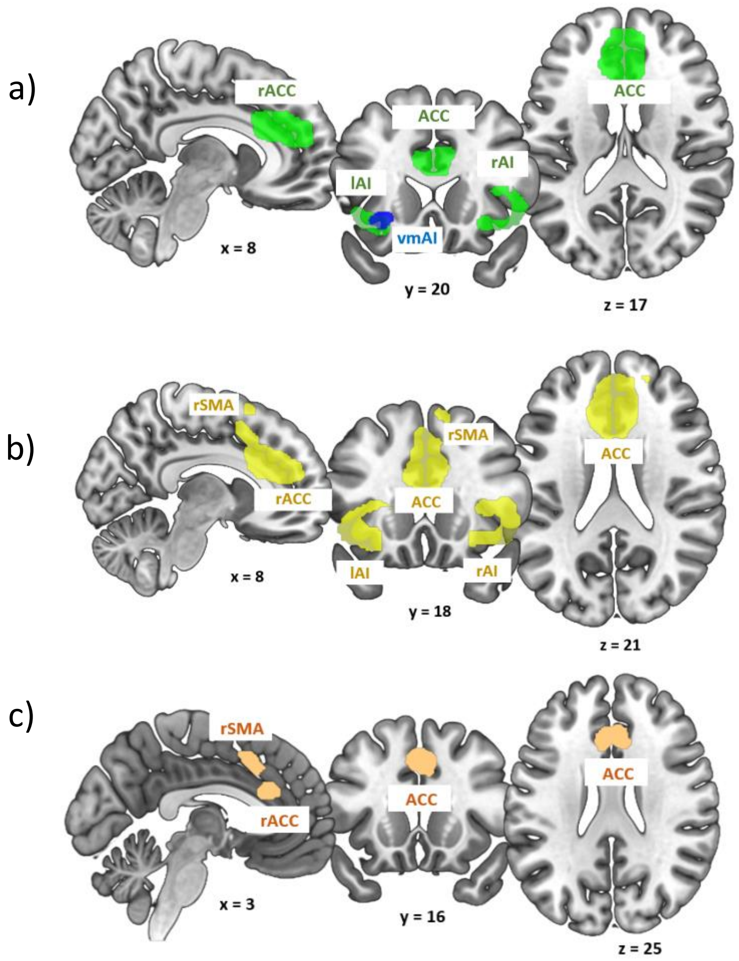

Figure S2. Sagittal, occipital, and axial slices depicting activation for different contrasts in GLM2. a) Main effect of self-gain lies vs. both other-gain and self-gain truths in green and the interaction effect with reputation risk in blue (Rep_SG-Lie + NoRep_SG/OG_Truth > Rep_SG/OG-Truth + NoRep_SG-Lie, b) Main effect of self-gain lies vs. self-gain truths, c) Main effect of other-gain truths vs. self-gain truths. No interaction effects were found with reputation risk for contrast b and c.

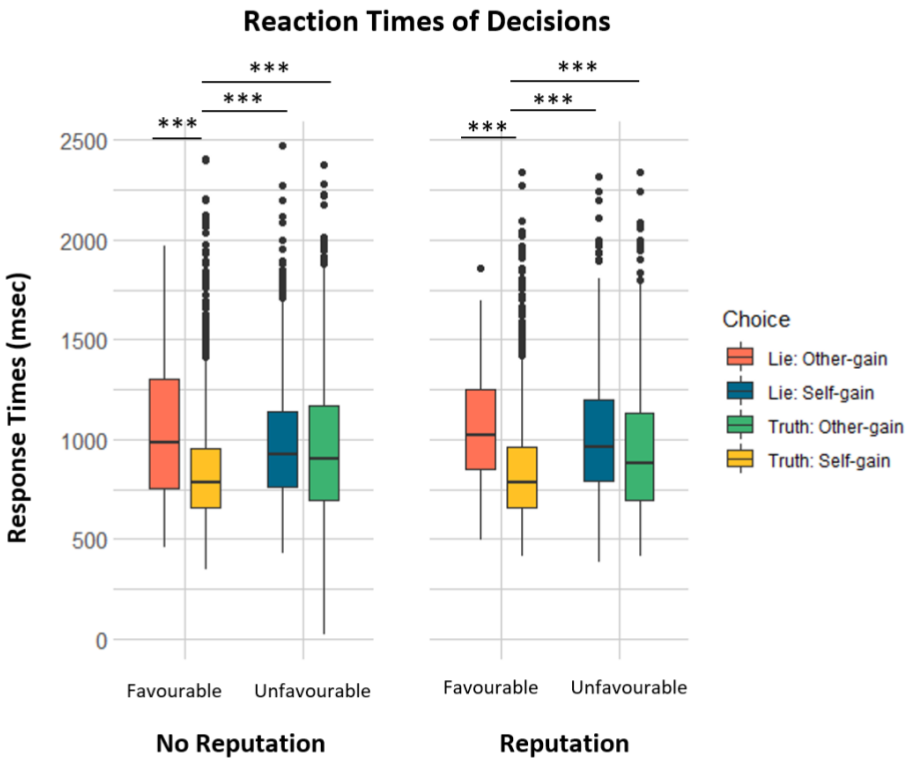

Figure S3. Boxplots of Reaction Times (RT) of the decisions for each reputation condition (No Rep or Rep) and for each outcome (Fav or UnFav). NoRep: SG-Truth vs. OG-Lie: z-ratio = -4.55, p = .0001, SG-Truth vs. SG-Lie: z-ratio = -4.48, p = .0002, SG-Truth vs. OG-Truth: z-ratio = 4.37, p = .0003; Rep: SG-Truth vs. OG-Lie: z-ratio = -4.62, p = .0001, SG-Truth vs. SG-Lie: z-ratio = -5.76, p < .0001, SG-Truth vs. OG-Truth: z-ratio = -4.17, p =.0008.

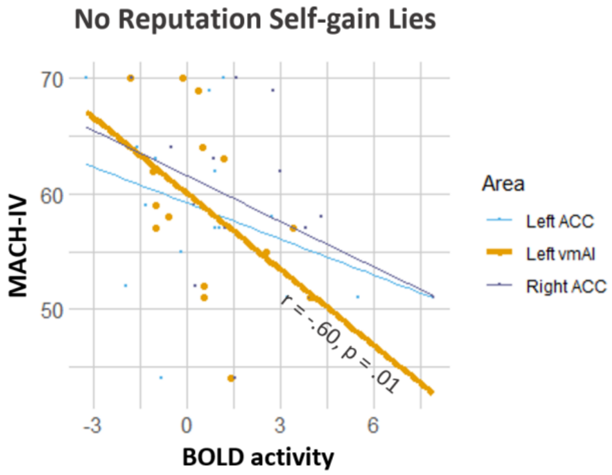

Figure S4. Correlation of averaged beta-values in 3 brain areas (LACC, LvmAI, RACC) during self-gain lies with no reputation risk and manipulativeness scores.

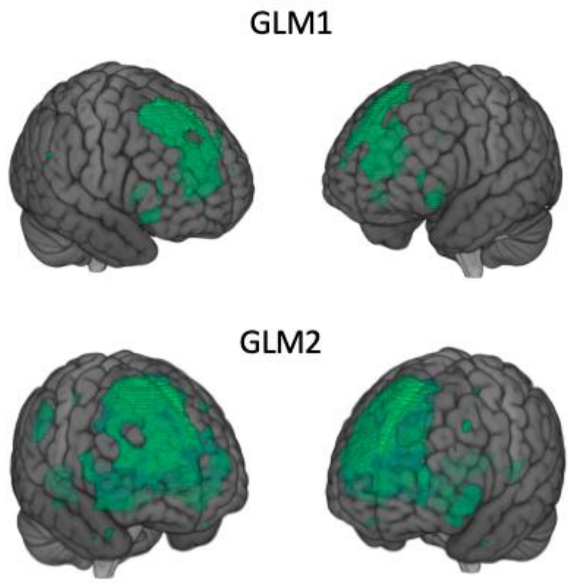

Figure S5. Whole-brain analysis using all spontaneous decision versus instructed conditions, thresholded with $p_{uncorrected}$ <.005. These volumes are used for Small Volume Correction in both GLM1 (Top) (N = 22) and GLM2 (Bottom) (N = 15).

Table S1. Trial count of each type of decision within each condition (no reputation or reputation) for GLM1. Participants are excluded when there were 6 trials or less within each condition. Encircled and underlined cells indicate counts of 6 or less and the 6 excluded participants.

Participant	No Reputation		Reputation	
	Lie	Truth	Lie	Truth
1	65	63	65	63
3	33	95	18	110
4	0	128	0	128
5	64	64	63	65
6	64	64	19	109
7	18	110	10	118
8	11	117	0	128
9	33	95	8	120
11	68	60	91	37
12	34	94	28	100
14	63	65	64	64
16	36	92	29	99
17	23	105	10	118
18	60	68	1	127
20	30	98	28	100
21	9	119	0	128
22	58	70	7	121
24	62	66	9	119
25	60	68	7	121
26	44	84	31	97
27	61	67	12	116
28	37	91	24	104
29	0	128	0	128
30	21	107	16	112
31	1	127	2	126
32	43	53	36	60
33	25	103	13	115
34	64	64	64	64

Table S2. Trial count of each type of decision within each condition (no reputation or reputation) for GLM2. Participants are excluded when there were 3 trials or less within each condition. Encircled and underlined cells indicate counts of 3 or less and the 7 excluded participants.

Participant	No Reputation			Reputation		
	SG-Lie	OG-Truth	SG-Truth	SG-Lie	OG-Truth	SG-Truth
1	62	2	61	64	0	63
3	28	36	59	13	51	59
5	64	0	64	63	1	64
6	64	0	64	19	45	64
7	18	46	64	10	54	64
9	32	32	63	8	56	64
11	31	33	27	47	17	20
12	34	30	64	28	36	64
14	63	1	64	62	2	62
16	25	39	53	20	44	55
17	23	41	64	9	55	63
20	30	34	64	28	36	64
22	58	6	64	4	60	63
24	61	3	63	8	56	63
25	60	4	64	6	58	64
26	23	41	43	17	47	50
27	61	3	64	12	52	64
28	27	37	54	13	51	53
30	18	46	61	16	48	64
32	32	16	37	28	20	40
33	16	48	55	10	54	61
34	64	0	64	64	0	64

Table S3. ROIs chosen for the connectivity analyses. Based on the main effect and interaction effect of interest (for the unfavourable outcome) and the main effect of lies vs. truths in GLM2 (see Table S4)

L/R	Regions	x	y	z	Contrast
R	ACC	8	48	20	ME: SG-Lie > OG-Truth
L	vmAI	-28	18	-6	Int: SG-Lie/OG-Truth x Rep/NoRep
R	ACC	8	28	30	Int: SG-Lie/OG-Truth x Rep/NoRep
L	ACC	-8	40	16	Int: SG-Lie/OG-Truth x Rep/NoRep
R	ACC	8	32	26	Int: SG-Lie/OG-Truth x Rep/NoRep
L	ACC	-4	26	22	ME: SG-Lie > OG+SG-Truth
L	ACC	-8	32	22	ME: SG-Lie > OG+SG-Truth
R	ACC	6	46	18	ME: SG-Lie > OG+SG-Truth
R	ACC	6	40	18	ME: SG-Lie > OG+SG-Truth
R	ACC	6	26	22	ME: SG-Lie > OG+SG-Truth
R	ACC	8	36	20	ME: SG-Lie > OG+SG-Truth
L	ACC	-6	42	16	ME: SG-Lie > OG+SG-Truth
L	dmPFC	-6	42	26	ME: SG-Lie > OG+SG-Truth
L	AI	-32	18	-10	ME: SG-Lie > OG+SG-Truth
L	AI	-40	12	-8	ME: SG-Lie > OG+SG-Truth
R	AI	30	20	-10	ME: SG-Lie > OG+SG-Truth

L/R, left/right; ACC, anterior cingulate cortex; dmPFC, dorsomedial prefrontal cortex; AI, anterior insula; vmAI, ventromedial anterior insula.

Table S4. Brain areas activated for different contrasts in GLM2. 1a) Main effect of self-gain lies vs other-gain and self-gain truths, 1b) interaction effect of self-gain lies vs other-gain and self-gain truths with reputation risk (Rep_SG-Lie + NoRep_SG/OG_Truth > Rep_SG/Og-Truth + NoRep_SG-Lie, 2a) self-gain lies vs. self-gain truths, 2) no interaction effect with reputation risk, 3a) other-gain truths vs. self-gain truths, 3b) no interaction effect with reputation risk. Only clusters and their local maxima that survived family-wise error correction (p <.05) are presented.

	L/R	Regions	kE	x	y	z	Z	P-FWE-corrected
1a) ME: SG-Lie > OG-Truth + SG-Truth								
	L	ACC	2318	-4	26	22	5.98	<0.001
	L	ACC		-8	32	22	5.4	<0.001
	R	ACC		6	46	18	5.33	<0.001
	R	ACC		6	40	18	5.16	0.001
	R	ACC		6	26	22	5.12	0.001
	R	ACC		8	36	20	5	0.001
	L	ACC		-6	42	16	4.95	0.002
	L	dmPFC		-6	42	26	4.67	0.006
	L	AI	392	-32	18	-10	4.74	0.005
	L	AI		-40	12	-8	4.61	0.008
	R	AI	299	30	20	-10	4.42	0.018
1b) Interaction with reputation								
	L	vmAI	141	-28	18	-6	4.28	0.031
2a) SG-Lie > SG-Truth	L	ACC	2912	-4	26	22	6.93	<0.001
	R	ACC		8	36	20	5.76	<0.001
				0	44	18	5.09	0.001
				0	18	44	4.97	0.002
	R	SMA		6	12	46	4.56	0.01
	L	AI	578	-34	18	-8	5.17	0.001
	L	AI		-40	14	-6	5.05	0.001
	R	AI	593	28	20	-10	5.02	0.001
	R	AI		44	16	-8	4.6	0.009
	R	Cerebral		36	20	8	4.39	0.02
2b) No interaction with reputation								
3a) OG-Truth > SG-Truth	R	SMA	797	4	14	44	4.94	0.002
	L	ACC		-2	30	26	4.22	0.039
	R	ACC		4	26	28	4.22	0.039
	L	ACC		-6	28	24	4.22	0.04
	R	ACC		6	28	24	4.19	0.045
3b) No interaction with reputation								

L/R, left/right; ACC, anterior cingulate cortex; dlPFC, dorsolateral prefrontal cortex; AI, anterior insula; vmAI, ventromedial anterior insula; SMA, supplementary motor area; SG-Lie, self-gain lie; OG-Truth, other-gain truth; SG-Truth, self-gain truth.

Table S5. Whole-brain analysis of the main effect (Lie > Truth) of GLM1. Peak-level Family-wise corrected p-values are given.

GLM1	L/M/R	Regions	kE	x	y	z	Z	P-FWE-corrected
ME: Lie > Truth	R	ACC	2647	4	28	28	5.69	<0.001
	L	ACC		-6	28	28	5.32	0.002
	R	ACC		6	44	20	4.75	0.027
	L	AI	685	-34	22	-8	5.52	0.001
	L	AI		-40	16	-8	5.1	0.006
	R	AI	432	30	18	-10	4.76	0.025

L/R, left/right; ACC, anterior cingulate cortex; AI, anterior insula

Table S6. Whole-brain analysis of the interaction effect (Rep_Lie/NoRep_Truth > NoRep_Lie/Rep_Truth). The interaction effect did not survive peak-level family-wise correction and cluster-level family-wise error correction, so cluster-level uncorrected p-values are given.

	L/M/R	Regions	kE	x	y	z	Z	P-uncorrected
INT	L	AI	117	-32	22	-10	4,04	0.031

L/R, left/right; AI, anterior insula

Table S7. Whole-brain analysis of the main effect (SG-Lie > SG-Truth/OG-Truth) of GLM1. Peak-level family-wise corrected p-values are given.

GLM2	L/M/R	Regions	kE	x	y	z	Z	P-FWE-corrected
ME	L	ACC	2721	-4	26	22	5,98	<0.001
	R	ACC		6	46	18	5.33	0.002
	R	ACC		6	26	22	5.12	0.005
	L	AI	413	-32	18	-10	4.74	0.027
	L	AI		-40	12	-8	4.61	0.046

L/R, left/right; ACC, anterior cingulate cortex; AI, anterior insula

Table S8. Whole-brain analysis of the interaction effect (Rep_SG-Lie/NoRep_OG-Truth > NoRep_SG-Lie/Rep_OG-Truth). The interaction effect did not survive peak-level family-wise error correction and cluster-level FWE-correction, so cluster-level uncorrected p-values are given.

	L/M/R	Regions	kE	x	y	z	Z	P-FWE-corrected
INT	L	dmPFC	1877	-10	58	14	4,47	<0.001
	R	ACC		8	28	30	4,21	<0.001
	L	ACC		-8	40	16	4,18	<0.001
	L	AI	314	-28	18	-6	4,42	0.01
		vlPFC		-46	16	8	3,85	0.01
	L	AI		-46	12	-2	3,46	0.01

L/R, left/right; dmPFC, dorsomedial prefrontal cortex; ACC, anterior cingulate cortex; AI, anterior insula; vlPFC: ventromedial prefrontal cortex; SG-Lie, self-gain lie; OG-Truth, other-gain truth

Q1. Questionnaire administered right after the scanning session

- How sure are you about the gender of the opponent from 1 to 10?

- How involved did you feel in the game from 1 to 10?

- How rightful do you think you were from 1 to 10?

- How guilty do you feel for lying from 1 to 10 when you could decide whether to tell the truth?

- How guilty do you feel for lying from 1 to 10 when you were obliged to do so by the instruction

- How guilty do you feel for lying from 1 to 10 when you knew the opponent could know (beta condition)?

- How guilty do you feel for lying from 1 to 10 when you knew the opponent could not know (lambda condition)?

- From 1 to 10, how much do you think your response is anonymous in the beta condition?

- From 1 to 10, how much do you think your answer is anonymous in the lambda condition?

- Do you regret not lying enough?

If yes, please indicate the condition in which you regret not having lied

- How much do you think you lied from 1 to 10?

- How much would you like to meet the opponent from 1 to 10?

- How sorry do you think the opponent is for you?

- How worried do you feel about meeting the opponent from 1 to 10?

- Can you explain in a few words why you lied to the opponent?

- Can you explain in a few words why you told the truth to the opponent?

- Do you think the opponent is male or female?
