# Supplementary material for: Reputation risk during dishonest social decision-making modulates anterior insular and cingulate cortex activity and connectivity
Source: Commun Biol. 2023 Apr 29;6:475. doi: 10.1038/s42003-023-04827-w (PMC10148859; doi:10.1038/s42003-023-04827-w)
Supplement: Supplementary file 2 — Reporting Summary [file 42003_2023_4827_MOESM2_ESM.pdf]

nature portfolio

Corresponding author(s): Salvatore Maria Aglioti
Last updated by author(s): 26/2/22

Reporting Summary

Nature Portfolio wishes to improve the reproducibility of the work that we publish. This form provides structure for consistency and transparency in reporting. For further information on Nature Portfolio policies, see our Editorial Policies and the Editorial Policy Checklist.

Statistics

For all statistical analyses, confirm that the following items are present in the figure legend, table legend, main text, or Methods section.

n/a	Confirmed	
☐	☒	The exact sample size (*n*) for each experimental group/condition, given as a discrete number and unit of measurement
☐	☒	A statement on whether measurements were taken from distinct samples or whether the same sample was measured repeatedly
☐	☒	The statistical test(s) used AND whether they are one- or two-sided<br>*Only common tests should be described solely by name; describe more complex techniques in the Methods section.*
☐	☒	A description of all covariates tested
☐	☒	A description of any assumptions or corrections, such as tests of normality and adjustment for multiple comparisons
☐	☒	A full description of the statistical parameters including central tendency (e.g. means) or other basic estimates (e.g. regression coefficient) AND variation (e.g. standard deviation) or associated estimates of uncertainty (e.g. confidence intervals)
☐	☒	For null hypothesis testing, the test statistic (e.g. *F*, *t*, *r*) with confidence intervals, effect sizes, degrees of freedom and *P* value noted<br>*Give P values as exact values whenever suitable.*
☒	☐	For Bayesian analysis, information on the choice of priors and Markov chain Monte Carlo settings
☐	☒	For hierarchical and complex designs, identification of the appropriate level for tests and full reporting of outcomes
☐	☒	Estimates of effect sizes (e.g. Cohen's *d*, Pearson's *r*), indicating how they were calculated

Our web collection on statistics for biologists contains articles on many of the points above.

Software and code

Policy information about availability of computer code

Data collection	For data collection, MATLAB R2019b was used. The MATLAB code for running the experiment and for extracting trial-by-trial data can be found here: https://doi.org/10.17605/OSF.IO/YK3NX.
Data analysis	R./RStudio was used for data analysis. The R. code and the corresponding excel data matrix can be found here: https://doi.org/10.17605/OSF.IO/DX8JU. MATLAB code for extracting onsets for fMRI data analysis can be found here: https://doi.org/10.17605/OSF.IO/ZVKNC

For manuscripts utilizing custom algorithms or software that are central to the research but not yet described in published literature, software must be made available to editors and reviewers. We strongly encourage code deposition in a community repository (e.g. GitHub). See the Nature Portfolio guidelines for submitting code & software for further information.

Data

Policy information about availability of data

All manuscripts must include a data availability statement. This statement should provide the following information, where applicable:

- Accession codes, unique identifiers, or web links for publicly available datasets
- A description of any restrictions on data availability
- For clinical datasets or third party data, please ensure that the statement adheres to our policy

The fMRI datasets of both models (source data: betas and contrast nifty files and SPM.mat files) (https://doi.org/10.17605/OSF.IO/ZVKNC) and behavioral data

(source data: excel behavioral data matrix) (https://doi.org/10.17605/OSF.IO/DX8JU) that support the findings of this study are available as a repository on the Open Science Framework: https://osf.io/v2jpn/). The repository also contains the raw MATLAB experimental output files, scripts to run the experiment, extract trial-by-trial data and extract onsets and the stimuli images (https://doi.org/10.17605/OSF.IO/YK3NX). Raw fMRI data, preprocessed nifti files and first-level SPM data will be made available upon request.

Human research participants

Policy information about studies involving human research participants and Sex and Gender in Research.

Reporting on sex and gender	We did not consider sex or gender differences in our study. Our aim was to look at a gender-balanced sample of the population. Notwithstanding that, following a Reviewer's request, we did run our analyses with gender as covariates. However, our findings indicated that none of the demographic factors had a significant effect on the results and were, thus, excluded from the analysis.
Population characteristics	Voluntary participants that were free from any contraindication to the MRI, and had no history of major psychiatric or neurological problems.
Recruitment	Participants were recruited using notices on social media and on bulletin boards of researchers
Ethics oversight	The independent Ethics Committee of the Santa Lucia Foundation IRCCS (Scientific Institute for Research Hospitalization and Health Care) approved the protocol

Note that full information on the approval of the study protocol must also be provided in the manuscript.

Field-specific reporting

Please select the one below that is the best fit for your research. If you are not sure, read the appropriate sections before making your selection.

☐ Life sciences ☒ Behavioural & social sciences ☐ Ecological, evolutionary & environmental sciences

For a reference copy of the document with all sections, see nature.com/documents/nr-reporting-summary-flat.pdf

Behavioural & social sciences study design

All studies must disclose on these points even when the disclosure is negative.

Study description	Our study was a mixed-methods study. Both quantitative and qualitative data were collected.
Research sample	Eighteen females and sixteen males were recruited. All participants had normal or corrected-to-normal vision, were free from any contraindication to the MRI, and had no history of major psychiatric or neurological problems.
Sampling strategy	The appropriate sample size for this study was estimated with G*Power 3.1.9.2 (ANOVA, repeated measures, within factors), considering a medium effect size of 0.20 (predicted based on Panasiti et al., 201126, using the same design), a significance level of 0.05, 1 group, 12 measurements (i.e., 3 reputation condition x 2 decisions x 2 outcomes). This indicated a power > 95% using a sample size of 28 participants.
Data collection	A Siemens Allegra fMRI scanner was used to collect the imaging data. While in the scanner, behavioral choices were recorded using a MATLAB script. Both a technician, blind to the conditions and study hypothesis, and a researcher, not blind to the conditions and study hypothesis was present during the data collections
Timing	Data were collected from 19/09/2012 until 31/01/2013
Data exclusions	Data from six participants were excluded prior to analyses (three participants were excluded due to technical problems related to the acquisition of the anatomical scan; two did not believe that the opponent was a real player; and one did not understand the task properly). For the fMRI analysis and for the first general linear model, data from 6 participants were excluded from the analyses due to insufficient trials, based on a criterion of less than 10% for lying or truth-telling decisions per condition per outcome. For the second general linear model, data from 13 participants were excluded based on the 10% criterion per decision (Self-gain Lie, Other-gain Truth, Self-Gain Truth) within each condition and outcome.
Non-participation	No participant has dropped out.
Randomization	Participants were not allocated to groups.

Reporting for specific materials, systems and methods

We require information from authors about some types of materials, experimental systems and methods used in many studies. Here, indicate whether each material, system or method listed is relevant to your study. If you are not sure if a list item applies to your research, read the appropriate section before selecting a response.

Materials & experimental systems

n/a	Involved in the study
☒	☐ Antibodies
☒	☐ Eukaryotic cell lines
☒	☐ Palaeontology and archaeology
☒	☐ Animals and other organisms
☒	☐ Clinical data
☒	☐ Dual use research of concern

Methods

n/a	Involved in the study
☒	☐ ChIP-seq
☒	☐ Flow cytometry
☐	☒ MRI-based neuroimaging

Magnetic resonance imaging

Experimental design

Design type	An event-related design was used.
Design specifications	There was a total of 384 trials, given by the crossing of 2 (left vs. right card selected by OP) by 2 (favorable vs. unfavorable outcome for participants, i.e., OP chose ace of spades or ace of hearts, respectively) by 3 (No Rep, Rep, or Ins experimental conditions) by 32 repetitions of each type of trial. The total experiment consisted of 4 functional MRI runs including 96 trials each. These were administered in 9 mini-blocks, 3 for each condition (i.e., No rep, Rep, or Ins), with different lengths, namely, either 8, 10 or 14 trials, to avoid predictability of the number of consecutive trials.
Behavioral performance measures	Number of the trials, the time of the start of the trial, the onset of the decision phase, the time of the the key pressed, the right or left key, the type of choice it was, the response times, the image used and the block-type.

Acquisition

Imaging type(s)	Functional and structural images
Field strength	3T
Sequence & imaging parameters	functional MR images were acquired using echo-planar imaging (EPI) [slices = 32, TR = 2.08 s, TE = 30 ms, in-plane resolution = 3 x 3 mm2, slice thickness = 2.5 mm, flip angle = 70 degrees), covering the entire cortex. Structural MR images were obtained using a T1-weighted 3D magnetization prepared rapid gradient echo (MPRAGE) imaging sequence [slices = 176, TR = 2s, TE = 4.38 ms, in-plane resolution = 0.5 x 0.5 mm2, slice thickness = 1mm, flip angle = 8 degrees]. For each participant, we acquired 1284 fMRI volumes, 321 for each of the four functional runs.
Area of acquisition	Whole brain scans were used. Yet, the first four volumes of each run were used for stabilizing longitudinal magnetization and then discarded from further analysis.

Diffusion MRI ☐ Used ☒ Not used

Preprocessing

Preprocessing software	The Statistical Parametric Mapping package SPM12 was used. Functional images were slice-time corrected, to compensate for slice acquisition delays between the first and last slice by using the middle slice as a reference, realigned and unwarped, to correct for head movement, registered to the first volume using a 2nd degree B-spline interpolation, whereas during the unwarp re-slicing a 4th degree B-spline interpolation was used. Individual structural T1-weighted images were segmented into grey matter (GM), white matter (WM) and cerebrospinal fluid (CSF) using SPM tissue probability maps. Structural images were bias-corrected with a light regularization and a 60 mm cut-off, while forward deformation fields were created. The segmented structural bias-corrected image was then co-registered with the slice-timed, realigned, and unwarped functional images, using the mean unwarped image as the source image. Images were smoothed with a Gaussian kernel of 8 mm FWHM to ameliorate differences in inter-subject localization.
Normalization	The obtained forward deformation fields during segmentation were used during normalization to bring co-registered functional images of the 4 sessions into the MNI space (2 mm isotropic voxel) using the 4th-degree B-Spline interpolation.
Normalization template	MNI
Noise and artifact removal	Using the ARTrepair toolbox, all images were previewed (art_movie) for detection of excessive motion artifacts and bad slices were detected and repaired (art_slice) by interpolating adjacent slices. After pre-processing, images were then examined again (art_detect) for detecting those scans in which the excessive motion remained. Outlier scans were identified in the temporal differences series by assessing between-scan differences (Z-threshold: 3.0 mm, scan-to-scan movement threshold: 1 mm; rotation threshold: 0.02 radians). The outlier scans (3.1% overall) were omitted from the analysis by including a single regressor for each outlier in the design matrix.

Volume censoring | See above

Statistical modeling & inference

Model type and settings: Univariate analysis was conducted, using first-level multiple regression models and second-level factorial design with subject as random factor.

Effect(s) tested: Two models were used; the first was a 3 (no reputation risk, reputation risk and instruction) x 2 (lie and truth) factorial design and the second was a 3 (no reputation risk, reputation risk and instruction) x 3 (self-gain lie, other-gain truth and self-gain truth) factorial design. Main effects included lies > truths and interaction effects with reputation conditions were tested.

Specify type of analysis: ☐ Whole brain ☐ ROI-based ☒ Both

Anatomical location(s): Small volume corrections were used to create regions of interest by using a thresholded contrast image of p-uncorrected < .005, contrasting all spontaneous conditions (reputation and no reputation) with instructed conditions.

Statistic type for inference (See Eklund et al. 2016): Data are presented including all significant activations at cluster-level. Additionally, local maxima in the clusters were included at peak-level.

Correction: A family-wise error (FWE) correction was used with p < .05

Models & analysis

n/a	Involved in the study
☐	☒ Functional and/or effective connectivity
☒	☐ Graph analysis
☒	☐ Multivariate modeling or predictive analysis

Functional and/or effective connectivity: The generalized psycho-physical interaction (gPPI) toolbox was used (CONN)
